# Nanotechnology in the Restoration of Polluted Soil

**DOI:** 10.3390/nano12050769

**Published:** 2022-02-24

**Authors:** Vishnu D. Rajput, Tatiana Minkina, Sudhir K. Upadhyay, Arpna Kumari, Anuj Ranjan, Saglara Mandzhieva, Svetlana Sushkova, Rupesh Kumar Singh, Krishan K. Verma

**Affiliations:** 1 Academy of Biology and Biotechnology, Southern Federal University, 344090 Rostov-on-Don, Russia; tminkina@mail.ru (T.M.); kumari@sfedu.ru (A.K.); randzhan@sfedu.ru (A.R.); msaglara@mail.ru (S.M.); terra_rossa@mail.ru (S.S.); 2 Department of Environmental Science, V.B.S. Purvanhal University, Jaunpur 222003, India; sku.env.lko@gmail.com; 3 InnovPlantProtect Collaborative Laboratory, Department of Protection of Specific Crops, 7350-999 Elvas, Portugal; rupeshbio702@gmail.com; 4 Guangxi Academy of Agricultural Sciences, Nanning 530007, China; drvermakishan@gmail.com

**Keywords:** pollution, heavy metals and metalloids, phytoremediation potential, phytorestoration strategy, nanotechnology

## Abstract

The advancements in nanoparticles (NPs) may be lighting the sustainable and eco-friendly path to accelerate the removal of toxic compounds from contaminated soils. Many efforts have been made to increase the efficiency of phytoremediation, such as the inclusion of chemical additives, the application of rhizobacteria, genetic engineering, etc. In this context, the integration of nanotechnology with bioremediation has introduced new dimensions for revamping the remediation methods. Hence, advanced remediation approaches combine nanotechnological and biological remediation methods in which the nanoscale process regulation supports the adsorption and deterioration of pollutants. Nanoparticles absorb/adsorb a large variety of contaminants and also catalyze reactions by lowering the energy required to break them down, owing to their unique surface properties. As a result, this remediation process reduces the accumulation of pollutants while limiting their spread from one medium to another. Therefore, this review article deals with all possibilities for the application of NPs for the remediation of contaminated soils and associated environmental concerns.

## 1. Introduction

Rapidly increasing anthropogenic/technogenic activities are adding potentially toxic metals, agrochemicals, and an excess of nutrients to the soil [1]. Soil is a basis of crop production as it supports plants to uptake nutrients [2]. In fact, agriculture sustains and defines human lives; however, it is often disruptive of natural ecosystems. Humans’ voracious appetites for getting the benefits from natural resources grow in tandem with population growth. The conflict between the benefits and the sustainable management of agricultural land and its conservation has been reported in the literature for a long time [3,4]. Land pollution is a threat to livelihoods, quality of life, and sustainable development [5]. Thus, conserving soil is the utmost requirement for the current era owing to the pressures of increasing population and the shrinking of arable lands by technogenic activities.

The advancements in nanotechnology open a window globally to remediate or restore polluted soil in an effective way [6,7]. It has been claimed that nanotechnology has great potential as an environmentally cleaner technology, including by alleviation of the toxicities of various metals/metalloids [8,9]. Besides, nanotechnology has been recognized as a potential method for the remediation of pollutants in a variety of environmental matrices, including soils [6]. In this context, soil remediation is one of the main domains where nanotechnological approaches have been widely used. The uses of NPs have been explored lately to remove contaminants in a variety of ways, including by adsorption, redox reactions, precipitation, and co-precipitation, all of which are aided by their enormous specific surface area [9].

With the help of NPs, hyperaccumulators and indigenous soil microbes could enhance biodegradation processes, thereby increasing the potential extent of remediation. This could be called nano-phytoremediation and microbial-mediated nano-remediation. The use of NPs with bioremediation approaches may result in a lot of benefits as these particles are small (1–100 nm) with a larger surface area and reactivity [10]. The broad range of studies indicated that the foliar use of NPs alleviates metal-pollutant toxicity and enhances plant growth, resulting in a high accumulation of elemental toxic content in plant tissues [11,12,13]. The degree of contamination, the bioavailability, and the accumulation of metals by the plants are decisive for the efficiency of nano-phytoremediation as a way of removing heavy metals (HMs) from contaminated sites [14,15].

The utilization of NPs based on a metal–organic framework (MOF) has received a lot of important attention lately, but it is primarily employed for drinking water and wastewater treatment [16,17,18]. Thus, there is a massive opportunity for scientists to envisage potential uses of MOF in soil remediation. However, concerns related to the safe use of NPs to remediate polluted soils, and their release into ecosystems, are still less explored, and this becomes a matter of concern [19,20]

The present review unbinds the possibilities for the elimination of contaminants, with particular emphasis on microbe-mediated remediation, hyperaccumulator plants, and NPs, as well as the approaches to restore HM-contaminated soils, the benefits, and the potential risks associated with nano-bioremediation technologies. The gaps and future perspectives are comprehensively elaborated.

## 2. An Appraisal of Nanobioremediation-Based Removal of Pollutants; Special Emphasis on Microbe-Mediated Remediation

Soil rich in vital nutrients and micronutrients is believed to best support the optimum health of the plant and its growth [21]. Human-made activities have currently polluted the soil with a variety of persistent organic compounds, viz., polyaromatic hydrocarbons (PAHs), polychlorinated biphenyls (PCBs), volatile organic compounds (VOCs), HMs (Hg and Pb), agrochemicals (pesticides, fungicides, and fertilizers), and also sometimes with excess nutrients [22]. At the same time, urbanization and industrialization have also added solid wastes, varieties of chemicals, and solvents to the environment and agricultural soil [23].

Nanobioremediation is a cost-effective technique of utilizing plants and microbes for the breakdown of pollutant compounds, ultimately improving soil quality and reducing pollution. By breaking down contaminants in the soil, the process may be able to eradicate, retain, or reduce the amount of pollutants present [24,25]. The efficiency of bioremediation has been studied and enhanced in the past using chemical additives or biotechnology [26] but nanotechnology further improved the process with a newer aspect [27]. A summary of different NP-mediated removal of pollutants from contaminated media is elaborated in Table 1.

Nanobioremediation implies both nanotechnology and bioremediation together, where the process is executed at the nanoscale. The target pollutants are adsorbed, degraded, or modified owing to the unique physicochemical properties of the NPs, which also act as catalysts and help to reduce the activation energy required for breaking down the compounds [28]. The nanobioremediation process has been explored and studied, and the most exploited NPs are carbon- and metal-based [29,30]. Polymeric NPs in the form of nanocapsules or nanospheres are also exceptional in the elimination of persistent pesticide compounds and long-chain hydrocarbons [31]. However, in the case of HMs, the challenge is entirely different as they are non-biodegradable, as well as very prone to entering biological systems and food chains [32].

**Figure 1:**
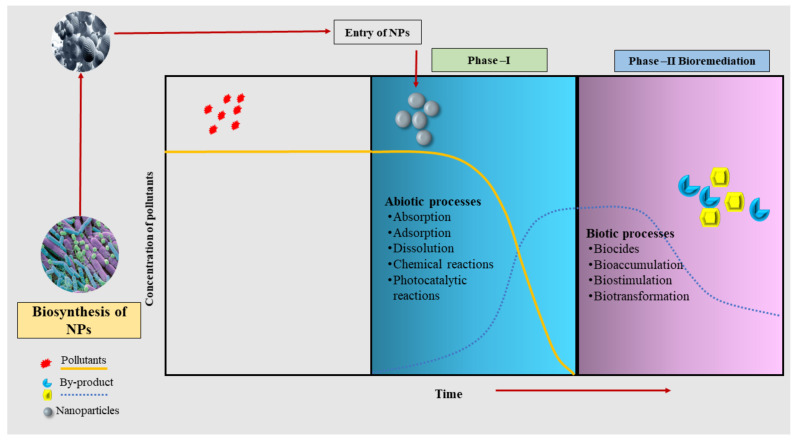
An overview of the processes of nanobioremediation using biogenic nanoparticles.

Biosorption and bioaccumulation using plants and microbes are traditional methods to remove HMs from polluted soils. However, recent pieces of evidence have reported the use of NPs in HM remediation with remarkable outcomes [33]. Nanoparticles are reported to have been applied in combination simultaneously or sequentially with specific microbes and the results have been convincing [34]. They could help to speed up the elimination of HMs by acting as nanocarriers of microbes or microbial biosorbents [35]. A pictorial diagram that represents the process of nanobioremediation, especially for biogenic NPs, is depicted in the Figure 1.

The synergy of NPs and bacterial degradation has also gained attention; however, the availability of a handful of published papers would not permit the making of a categorical review. Many authors have given their efforts to nanobioremediation, and yet it is still too insignificant for making any conclusive decision [10].

Integration of NPs with microbes for bioremediation is a two-phasic process that involves overlapping abiotic and biotic processes (Figure 1) [36]. In the first phase, after the entry of NPs into the system, pollutants undergo varieties of physicochemical processes and modifications depicting abiotic processes such as absorption, adsorption, dissolution, and chemical catalysis of photocatalytic reactions [37]. The second phase includes biotic processes such as biocides, bioaccumulation, biostimulation, and biotransformation [38,39]. These biotic processes play a crucial role in the removal of pollutants from the system.

### 2.1. Nanobioremediation of Heavy Metals

The existence of HMs in the environment is largely due to increased anthropogenic activities. However, disturbed biogeochemical cycles are also responsible for their release into the environment as pollutants. Elements like As, Cd, Cr, Hg, and Pb have no biological functions to perform in the biological system. Heavy metals comprise major inorganic pollutants as they exhibit substantial toxic impacts on biota even at the lowest concentrations [40,41]. The toxicity of HMs also rests on their bioavailability and absorption [42]. Acidic environments instigate the toxicity of HMs, especially if the soil structure is poor and has low nutrients (e.g., mining areas) [43].

Heavy metals primarily affect the plants and lower soil organisms by inducing the generation of reactive oxygen species (ROS), which further results in the damage of macromolecules such as proteins and nucleic acids [41]. The existence of HMs in the soil affects crops and vegetation, their nutritional quality, and the ecological aspects associated with them. The effect of HMs on crops varies depending on the crop species, soil physicochemical characteristics, and HM type [51]. The general mechanism of toxicity exerted by HMs on crop plants includes ROS generation, which affects the cell organelles, macromolecules such as proteins and nucleic acids, and other components of the plant’s structure and function [51,52]. It has also been reported to affect respiration and photosynthesis, reduce enzyme activities, elevate oxidative stress, reduce biomass, diminish crop yield, and affect the abundance, activity, diversity, and genetic makeups of useful soil microflora [53,54].

One of the key methods for the elimination of HMs includes site stabilization that immobilizes them at a specific site to decreases mobility and availability in the soil, and stops them from leaching across the sites [55]. The use of various NPs, including biogenic, has been gaining a lot of attention for the removal of HMs [56]. Biogenic NPs are those that are synthesized using biological organisms. The commonly known biogenic NPs, such as Ag NPs, are formed by *Morganella psychrotolerans* [57,58].

Nanoparticles of FeO coated with polyvinylpyrrolidone (PVP) have been successfully used for improving the bioremediation of the soil contaminated with Pb and Cd by a Gram-negative bacteria, *Halomonas* sp. This approach has significantly removed nearly 100% of Pb after 24 h, and Cd after 48 h, as compared to removal by bacteria or only NPs [59]. A biosorbent of magnetic Fe_3_O_4_ NPs treated with *S. aureus*, with a surface encapsulated with phthalic acid (as a n-Fe_3_O_4_-Phth-S complex), was used for the removal of Cu, Ni, and Pb, and the adsorptive removal of 795, 1355, and 985 µmol g^−1^ for Cu, Pb, and Ni was achieved, respectively. In terms of percentage, the recovery rates of 83.0–89.5% for Cu^2+^, 99.4–100% for Pb^2+^, and 92.6–7.5% for Ni^2+^ were observed. The comparative study with dried *S. aureus* and n-Fe_3_O_4_-Phth-S for HM removal inferred that the n-Fe_3_O_4_-Phth-S core of the NPs, as well as the functional groups present on the microbial surface, played a key role in the removal of HMs [49]. Thus, this work revealed that the core of the NPs, as well as functional groups present on the microbial surface, had an important impact on the elimination of the contaminants.

A recent study on the removal of Cu, Cd, Cr, and Pb using HM-resistant bacteria such as *B. cereus* (PMBL-3) and *L. macroides* (PMBL-7) evidently confirmed that ZnO NPs at 5 mg L^−1^ synergistically removes the Cr by 60%, the Cu by 70%, and the Pb by 85%, as compared to *B. cereus* (80 and 60%) and *L. macroides* (55 and 50%) at neutral pH, respectively [46]. At neutral pH the surface of ZnO NPs exhibit negative charges that promote electrostatic interactions with metal cations; however, at lower pH, the HMs get precipitated as hydroxides and then hydrogen ions compete for binding with adsorbents [60]. The strain XMCr-6 of *B.* *cereus* has also been reported to reduce the Cr^6+^ through an enzyme-mediated process. The reduced Cr^3^^+^ was observed to have a binding affinity to cells using coordination bonds with the functional group present on the surface of the bacterial cell wall. The formation of Cr_2_O_3_ NPs was found on the cell surface as a by-product [61].

The use of probiotic bacteria (*L. casei* and *L. fermentum*) to absorb Cd from water in association with Se^5+^ and Se NPs was also investigated. The higher absorption of Cd by *L. casei* with Se^4+^ ions (65%), compared to Se NPs (55.90%), was discovered in this study, and it was correlated to the higher solubility of Se^5+^ compared to Se NPs. When comparing *L. fermentum* and *L. casei*, the efficiency of Cd absorption was significantly higher in *L. fermentum* (50.87%) than *L. casei* (43.78%). The percentage of Cd adsorption by *L. casei* when used in conjunction with Se NPs shows no significant change. However, with increased Se NPs ratio percentages, Cd absorption was slightly increased from 5.49 to 16.54 in the presence of *L. casei* with Se NPs, compared to *L. casei* [62].

A threefold approach is now gaining popularity as the HM pollutants can be used by selective microbes to synthesize biogenic NPs (resource recovery), thereby removing them from the environment (remediation) and yielding value for the waste (effective waste utilization). A study using *Enterococcus faecalis* for biorecovery of Pd as Pd NPs reported the synthesis of intra- and extra-cellular (membrane-bound). The range of Pd NPs was observed as 10 nm by transmission electron microscopy; however, the size of the Pd NPs was dependent on environmental conditions such as temperature, pH, and biomass. The obtained Pd NPs have great use as a bionanocatalyst that shows good catalytic efficiency (6.3 mg Pd NPs completely reduced 5.0 µmol Cr^6+^ in 12 h) and the application is potentially useful to treat industrial effluents [44].

A similar study produced Te NPs from anaerobic sludge based upon supplementation with riboflavin [63]. It formed insoluble elemental tellurium (Te^0^ NPs) using pollutant tellurite Te^4+^ oxyanions present in the wastewater. It has been reported that 2-Hydroxy-1,4-naphthoquinone promotes the reduction of Te^4+^ and the quantity of Te^0^NPs synthesis [64]. The process is supported by *Rhodobacter capsulatus*, where malate is the electron-donating substrate [65], and riboflavin speeds up the rate of Te^4+^ reduction by anaerobic methanogenic granular sludge [66].

### 2.2. Degradation of Persistent Organic Pollutants

The pollution posed by POPs has been shown to have a negative influence on both the environment and human health, as certain POPs have been found to bioaccumulate in adipose tissue and to have the potential to act as carcinogens. Therefore, their remediation is a major challenge and is obligatory. A Gram-negative bacterial strain (NM05 of *Sphingomonas*) was earlier reported to degrade the pesticide hexachlorocyclohexane (HCH) [67] upon treatment with Pd/Fe0 bimetallic NPs (CMC-Pd/nFe0), showing the synergistic effect on the degradation of HCH that was enhanced by nearly 1.7–2.1-fold compared to the controls that had the *Sphingomonas* sp. strain NM05 or CMC-Pd/nFe0 alone [60]. The degradation process was found to be affected by experimental conditions (pH, temperature, HCH concentrations, etc.) [68].

The perovskite (LaFeO_3_) NPs and biochar from water caltrop (*Trapa natans*) shells studied on marine sediment reported enhanced degradation of PAHs. The study used lignocellulosic fiber-reinforced biodegradable composites (LFBC) at 0.75 g L^−1^ and pH 6.0 to activate the peroxymonosulfate (3 × 10^−4^ M) that helped in the oxidation of oxidizing PAHs in the sediments.

Up to 90% of total degradation was achieved; however, individually 2-ring PAHs 52%, 3-ring PAHs 61%, 4-ring PAHs 66%, 5-ring PAHs 56%, and 6-ring PAHs 29% were observed [69]. The process also reported improved microbial diversity of sediment and the major phylum *Proteobacteria* was observed initially, but after the process, *Hyphomonas* was predominantly observed [70]. In a continuous-flow experiments system for the degradation of naphthalene in the groundwater, 400 mg L^−1^ of synthesized CaO_2_ NPs degraded the naphthalene of optimum concentration 20 mg L^−1^. This study highlights complete remediation of naphthalene in the presence of CaO_2_ NPs and microbes (an abundance of *Coccobacilli*) from column effluent within 50 days [71].

In the case of soil, improving the microbial community by application of NPs is another way to reduce/remove the toxic pollutant loads from it. Si NPs have been reported to improve microbial colonization and biomass, including the rhizospheric microbes that are helpful for improving soil health [72,73]. However, prolonged exposure and accumulation of these NPs in soil may affect the nutrient and organic matter content.

## 3. Remediation of Contaminated Soils with Heavy Metals Using Hyperaccumulator Plants and Nanoparticles

Both from an ecological standpoint and one of restoring degraded areas, the remediation of HM-contaminated soils is a pressing issue that needs to be addressed immediately [74]. This section discusses the application of hyperaccumulation systems based on plants and NPs for the cleanup of various HMs from contaminated sites.

The kinds of soil found at a contaminated site, as well as the percentage of metal contamination present, influence the rate at which hyperaccumulating plants can be utilized to remediate the site [75]. The bioavailability of metals in the rhizospheric region rests on soil pH, the gradient in elemental concentration, microbial population change, redox potential, the ratio of CO_2_ and O_2_, etc. [76]. The rhizospheric environment also directly rests on plant species in terms of root exudates and root architecture [75].

A few plant species, such as *Pedioplanis burchelli*, *Amaranthus spinosus*, and *Alternanthera pungens*, have survived at an optimal level of HMs by rhizofiltration and demonstrated avoidance mechanisms for HM uptake in their environment [70]. Beyond the optimum concentration, HMs pose an adverse impact on plant growth and human health [77]. However, hyperaccumulating plant species ingest metals in large quantities from contaminated soils, then transport and accumulate them in the organs above the soil in higher concentrations than in non-hyperaccumulating species without any obvious phytotoxic effects [78,79]. The activity of plant hyperaccumulators for HMs based on their phytoremediation potentials such as phytostabilization, phytoextraction, and rhizodegradation was demonstrated [80].

**Figure 2:**
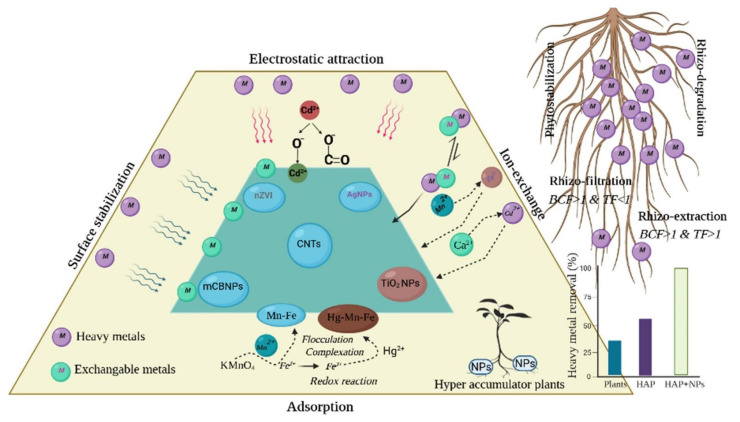
Schematic representation of hyperaccumulator plant mechanistic supplemented with nanoparticles for removal of heavy metals from contaminated soil.

The hyperaccumulator plant that has a bio-concentration factor (BCF) of HMs of more than one owes it to the mechanism of phytostabilization and phytoextraction [76]. A BCF and TF (translocation factor) of more than one shows the characteristic traits of phytostabilization [81]. Similarly, Kisku et al. [81] demonstrated both phytostabilization and phytoextraction activities could be found in *Parthenium hysterophorus*, *Sacrum munja*, and *Ipomoea carnea*, and the authors observed more than one BCF and TF for Cr, Ni, Cd, and Pb, which revealed a phytostabilization mechanism, while more than one BCF and less than one TF for Zn and Mn showed a phytoextraction process for HMs [82]. Schematic representations of hyperaccumulator plant mechanistic supplemented with NPs for the elimination of toxic elements from contaminated soil are presented in Figure 2.

Rhizodegradation is the process through which pollutants are deposited in the rhizospheric area of soil by microbial activity, where bacteria metabolize these contaminants for energy and nutrition. In this mechanism, microbes are capable of breaking down hazardous pollutants into nontoxic and harmless products [83]. Plant roots release natural carbon-containing substances, such as sugar, alcohol, and acid, and thereby provide the microorganisms with additional nutrients, which further stimulates rhizodegradation activities [84].

A wide range of treatment strategies, including physicochemical and biological methods, have been used to decontaminate sites polluted with HMs. The mechanisms of these methods are based on redox reactions, adsorption, ion-exchange, bioremediation, and phytoremediation [85]. All these methods have their own deserves and demerits, and out of these methods’ bioremediation have come the most suitable eco-friendly techniques to achieve the sustainable goals [86,87]. Phytoremediation is a commonly explored technique and its potential application in contaminated land can be manipulated by added material such as NPs. Out of numerous mechanisms, the adsorbent mechanism plays a crucial role in the elimination of a broad range of HMs from contaminated soil in a short time.

Recently, adsorbent materials such as activated carbon, biochar, and NPs became commonly available; these adsorbent materials exhibit rapid adsorption capacity, cover large surface areas, provide more interplay sites for HMs, and have low price value [88]. Hence, using hyperaccumulator plants supplemented with good adsorbent material can be a promising approach for the elimination of HMs from contaminated soil. According to the existing state of knowledge, NPs have considerable potential for the remediation of soils contaminated with HMs. Nanophytoremediation is a process for the remediation of pollutants, i.e., pollutants that use synthetic NPs from plants [89].

For the remediation of soil contaminated with HMs, carbon or non-carbon NPs have primarily been employed; CNTs (carbon nanotubes), nZVI (nano zero-valent iron), TiO_2_ NPs, and Ag NPs have all been extensively investigated for their potential for soil remediation [90]. The HM-removal efficiency of CNTs also relies on the pH and temperature of the adjacent surrounding environment, contact time, and the dose of CNTs. The CNTs (0.05 g) remove 99.9% of Zn^2+^ at 10.0 pH [84] and a high percent of Cd^2+^ are removed at 3.0 pH [77]; FeS NPs removed 99.65% of Cr^6+^ at 6.0 pH, while at high pH (>10.0) the FeS NPs elimination rate was decreased [91]. The equilibrium of the adsorption ability of NPs rests on temperature fluctuations. The adsorption of Pb^2+^ by Fe_3_O_4_ NPs rises from 298 to 328K [92], while chitosan-alginate NPs remove Hg^2+^ at 30 °C [93]. Hg^2+^ removal efficacy by FeS NPs (stabilized by sodium carboxymethyl cellulose) achieves its highest elimination percent at 30 min of contact time [94]. The nZVI-NPs are effective for the elimination of HMs from contaminated sites, nZVI contains shells of zerovalent iron, Fe^2+^ and Fe^3+^, which develop the excellent potential of nZVI for the elimination of HMs. Removal of Zn^2+^ (109.7 mg g^−1^), Cu^2+^ (161.9 mg g^−1^), and Pb^2+^ (195 mg g^−1^) was achieved by utilization of nZVI after 6 h [95].

Several HMs (Cr, Zn, Pb, As, U, V, etc.) are effectively removed by the application of nZVI at the highest rate from contaminated sites [96]. The nZVI @ BC (8 g kg^−1^) was utilized in the pot experiment, and it was observed that the immobilization efficiency of Cr^6+^ from the soil was increased by 100% after 15 days [77]. The elimination of Cd, Pb, and Zn from contaminated soils was increased by the application of the nanomaterial OA-nZVI (0.4 g kg^−1^) by 46.66%, 48.88%, and 47.01%, respectively, reported [97]. The low concentration of nZVI substantially improved seedlings, root length, and leaf area in white willow (*Salix alba* L.) with an increased bio-concentration factor for Cd, while a high concentration of nZVI showed an adverse effect on plant growth and BCF for Cu and Pb [98]. The elimination of Zn, Pb, and Cd was investigated by Mitzia et al. [99] using nZVI with biochar and they observed that both combinations significantly affect the immobilization of Zn, Pb, and Cd. The nZVI (sodium carboxymethyl cellulose stabilized) showed a significantly increased immobilization of Cr in edible rapeseed (*Brassica napus*) and Chinese cabbage (*B. rapa* subsp. pekinensis) [100]. The use of NPs decreased the bioavailability and bioaccumulation of Cr in both plants.

It is observed that TiO_2_ NPs have high reactivity and photo-catalytic activity, which enables contaminates to get adsorbed on its surface area; this trait of TiO_2_ NPs serves to minimize the toxic behavior of HMs, as well as maintain their mobility. It absorbed 88.01% of Cu^2+^ and 70.67% of Cd^2+^ effectively at 7.0 pH [47]. Other NPs such as modified carbon substantially improved the plant growth of *Suaeda salsa* and decreased the uptake of Cd and Ni compared to the control [101]. Ag NPs can effectively reduce Ni, Pb, Na, Zn, and Cu uptake in maize (*Zea mays*) plants and promote plant growth by optimizing the gibberellin, abscisic content in *Z. mays* leaves and PGPR interaction [102]. In combination with *B. cereus* (LPR2), Ag NPs significantly induce the growth of *Z. mays* [103]. Hence, perfect strategies are required for the elimination of HMs from contaminated soil, such as the selection of hyperaccumulator plants with compatible NPs, which could be a promising technique for the restoration of contaminated soil.

## 4. Nanotechnological Approaches for Restoring Metalloid-Contaminated Soils

Metalloids are elements with a plurality of attributes, i.e., they have the physicochemical features of both metals and nonmetals. They are naturally found, but the introduction of industrialization has resulted in concentrations that exceed the allowable limit [104]. Antimony (Sb), arsenic (As), boron (B), germanium (Ge), tellurium (Te), and silicon (Si) are the six elements of the periodic table that are generally known as metalloids [105]. Metalloid pollution of soil is a global issue owing to the deposition of these chemicals in the environment, affecting human health, plants, and animals. Also, they are hazardous at any concentration, even at extremely low levels [9,106]. Besides, HMs/metalloids can have a major effect on the abundance, structure, and diversity of soil microbial communities, which can lead to a shift in ecosystem functioning [107,108].

In previous years, several remediation methods have been introduced for the reclamation of these metalloids from soil. However, successful remediation of sites contaminated by metalloids is hindered by the fact that these pollutants do not decompose on their own and that it is not always possible to extract all of the contaminated soil [9]. Therefore, chemical stabilization of metalloids in soils, through adsorption, surface precipitation, structural integration, or ion exchange, is a promising solution for such sites since it immobilizes contaminants in the soils, limiting their mobility, bioavailability, and bio-accessibility [109]. Furthermore, it is evident that the initial phase in phytoremediation, the lowering of their toxicity, is critical for the creation of plant cover on contaminated soils [80].

Among NPs, iron oxides NPs are considered to remediate contaminants like HMs from soils owing to their capacity to absorb these pollutants [109]. In this context, in a study, Zhang et al. [110] revealed the effectiveness of iron-based NPs, viz., nZVI, FeS, and Fe_3_O_4_ particles for immobilizing As in contaminated soils. Among these, Fe_3_O_4_ NPs were reported to be more effective than other NPs for immobilizing As. Besides iron oxides, other NPs are also described as remediating soils polluted with different metals and metalloids [111]. Apart from magnetite, other famous iron NPs are nZVI, which are utilized for remediation of metal/metalloid-polluted water. In a study, the photolytic system was used for the decrease in reducing the toxic form of chromium Cr^6+^ to non-toxic Cr^3+^ in aqueous [112]. In the same study, the Cr^6+^ reduction efficiency was recorded up to 90%. Furthermore, in some cases, NPs were also employed to immobilize the trace elements in contaminated soil. For example, nanostructured TiO_2_, MgO, and ZnO were found to be efficient adsorbents for the elimination of Cr ions from leather factory waste treated soil [113]. Hence, these aspects can be further explored and investigated to envisage the potential of such NPs for the elimination of metalloids from polluted soils. The nanoscale amorphous MnO was also evaluated for its capacity to remediate the soils polluted with HMs such as Cd, Cu, Zn, As, and Pb [114,115]. Although different kinds of NPs are less generally used for metalloid remediation, but they can still be applied in this context because some NPs are utilized to remove HMs from other contaminated media, such as water.

## 5. Nanobioremediation: Environment Concerns and Fate of Nanoparticles

Despite the numerous roles of and advancements in nanotechnology, there is still concern about its presence in environmental spheres, its fate, and the consequent toxicological impacts. Therefore, the main focus of this section is to distinctly provide an overview on the fate and the emerging environmental challenges of the deliberate emission of NPs, owing to their enhanced applications, especially in remediation processes. Recently, some reports documented that the increasing applications of NPs in agriculture have imposed serious implications. For example, the deliberate application of NPs may result in their accumulation or in an increase in the concentration of their constituents in the soil, thus affecting the soil’s properties [8,20]. The presence of NPs in soils is reported to alter the soil pH, which is one of the most important parameters that influences soil nutrient availability, microbial dynamics, overall soil health, and plant growth and development [116].

A study conducted by Cullen reported that modification of nanoscale zero-valent iron (nZVI) in the soil can cause a significant rise in the pH of a soil solution [117]. A similar report was claimed by a study of CuO NPs on soil pH where CuO NPs utilize H^+^ from the soil to yield Cu ions and Cu(OH)+ ionic complex. Furthermore, this process is stated to be more enhanced in acidic soil [118]. Also, NPs of Ag, Au, Ti, and Zn have been reported to affect soil pH and their presence has been associated with adverse effects on beneficial soil microorganisms and nematodes [119]. The extent of the induced detrimental effects of the presence of NPs in soil is influenced by their concentration and type, soil type, and the enzymatic activity of the soil [120]. In addition, an elevated concentration of NPs is associated with decreased dehydrogenase activity, which disrupts the equilibrium of the soil nutrient and fertility levels [120,121]. Furthermore, the absorption and internalization of such NPs by microbes significantly affects mycelium and damages their normal cell functioning [122].

In soil, there are three main procedures, viz., surface complexation, hydrophobic partitioning, and ion exchange for the sorption of NPs, which are also involved in the colloid-facilitated transport of pollutants [123]. Mobilization processes mediated by colloids are also responsible for the likelihood of assisted transfer. It is possible for nanoscale particles to have a significant impact on trace metal transport, either by slowing it down when they are trapped in the matrix or by increasing the speed while they are moving [124]. The presence of NPs has been reported in water [125,126], in soil [127,128], and also in the air [129,130]. Continuous NP emissions into the environment would very probably result in their ubiquitous presence, with NPs likely to infiltrate the food chain at various trophic levels and exert toxicological effects on a variety of aquatic and terrestrial animals, as well as on human health [131,132]. The ecotoxicity of NPs is strongly related to their negative effect on the environment, where they might interact with biological systems because of their unique physicochemical properties [133,134].

Once NPs enter into water bodies, they act as pollutants and are ingested by lower aquatic biota. Metal NPs were tested in three different animal models representing different trophic levels, including the *Danio rero* (zebrafish), *Daphnia pulex* (daphnia), and *Pseudokirchneriella subcapitata* (microalgae), and they caused acute toxicity and filter-feeding, according to the findings of the study. It was noted that the NPs were more hazardous to daphnia and microalgae than to zebrafish, which is understandable because daphnids are particle filter feeders and, hence, are more susceptible to being exposed to NPs [135]. Thus, after entering the biological system, NPs have an impact on biochemical processes at the molecular and tissue-organ levels [136,137]. DNA damage, ROS-induced oxidative stress, protein folding disruption, and cell death are the most common toxicological effects [138,139,140]. Some studies also documented that SiO_2_ NPs and TiO_2_ NPs induced immunomodulatory and immunosuppressive effects in biological systems [141,142]. Thus, before the implementation of NPs for nanobioremediation, these environmental concerns should be considered as of the utmost importance and NPs should be designed in ways that promote sustainable applications.

## 6. Conclusions and Future of Nanoremediation

In this modern period, there is a rapid release of pollutants into the environment, posing a substantial hazard to both human and the environmental health. As a result, having effective techniques for removing them from various environmental media will be critical in preventing their negative impacts. Because many conventional procedures are incapable of efficiently removing different groups of contaminants, novel approaches are therefore required to eliminate pollutants to the maximum extent possible. Meanwhile, a very likely method for developing remedies for polluted media is nanotechnology, according to the observations from a profusion of studies in this respect. A favorable result is the filling of the data gap on real-world remediation, which is aided by closer collaboration between the research and the industry. Thus, it will be useful for driving future soil cleanup efforts, which are sometimes necessary and cannot wait for experimental results to be obtained, utilizing methods to be attained in the meanwhile. Besides, the majority of the currently available research on nanobioremediation is limited to laboratory experiments and computational modelling. Therefore, to alleviate soils contaminated with a varied range of contaminants in the field, it is necessary to utilize multidisciplinary approaches. However, the environmental fate of NPs should be carefully taken into consideration before widening the application of nanobioremediation approaches.

## Figures and Tables

**Table 1:** Summary of different nanoparticles-mediated removal of different pollutants from contaminated media.

Nanoparticles	Remediated Contaminant(s)	Operational Conditions and Removal Efficiency	References
Polyvinylpyrrolidone (PVP) coated iron oxide nanoparticles	Cd and Pb	NPs applications were integrated with the process of bioremediation mediated by *Halomonas* sp. In the removal setup of Cd and Pb, *Halomonas* sp. was inoculated for 48 h at 180 rpm, 28 °C. The 100% removal was recorded after 24 h, while for Cd, it was observed after 48 h.	[44]
Zero-valent iron (nZVI) commercial suspension at two doses (1% and 10%)	As	pH was set at 12.2 ± 0.1 of the nZVI suspension. To avoid the aggregation of nZVI in the suspension, polyacrylic acid was used as a stabilizer. Maximal immobilization of As in brownfield soil was recorded at 10% of nZVI.	[45]
Graphene oxide nanoparticles (nGOx) and nZVI	Metals, viz., Cd, Pb, Zn, Cu, and As in the As-Metals polluted soil	Applications of nZVI and nGOx to the polluted soils considerably impacted the availability of As and metals. Cu, Pb, and Cd were immobilized by nGOx, while mobilized As and P. In a turn of nZVI, it immobilized the effectively As and Pb, and poorly Cd but enhanced availability of Cu. This study revealed that both NPs applications might be act as strategies for the immobilization and stabilization that can later be utilized for phytoremediation.	[46]
Titanium oxide nanoparticles-bonded-chitosan nanolayer (NTiO_2_-NCh)	Cd and Cu	The pH was set at 7.0 during the experimentation. The removal was assisted by 60–70 s heating by using microwave–enforced sorption approach. Application of NTiO_2_-NCh was found to eliminate Cu and Cd by 88.01% and 70.67%, respectively.	[47]
Palladium (Pd), Pd NPs	Cr	The use Pd NPs as bionanocatalyst has been explored. It was found that Pd NPs completely reduced Cr^6+^ in 12 h. 6.3 mg of PdNPs was used to reduce 5.0 µmol of Cr^6+^.	[48]
Magnetic iron oxide nanoparticles (Fe_3_O_4_ NPs) treated with *Staphylococcus aureus*, and surface encapsulated with phthalic acid (n-Fe_3_O_4_-Phth-*S aureus*)	Cu, Ni, Pb	n-Fe_3_O_4_-Phth-S was found to remediate 83.0–89.5%, for Cu^2+^, 99.4–100%, for Pb^2+^, and 92.6–7.5% for Ni^2+^. The study also identified n-Fe_3_O_4_-Phth-*S. aureus* as an excellent biosorbent for the removal of divalent ions from an aqueous medium.	[49]
ZnO NPs	Cu, Cd, Cr, and Pb	The applications of ZnO-NPs at 5 mg L^−1^ with *Bacillus cereus* and *Lysinibacillus macroides* showed the maximal removal of Cr, Cu, and Pb which was 60%, 70%, and 85%, respectively. The optimal pH for efficient removal was 8.0. The removal was less in the case of bacteria-mediated remediation which was found to be 83 and 70% in *B. cereus* and 60 and 65% in *L. macroides*.	[50]

## Data Availability

Not Applicable.
